# Reward-Related Brain Function as an Endophenotype in the Mood-Psychosis Spectrum

**DOI:** 10.1016/j.bpsgos.2026.100692

**Published:** 2026-01-21

**Authors:** Marjolein E.A. Barendse, Robert M. Bilder, Anne L. Snijders, Marit I. Broer, Laura J. van den Brink-Steinmann, Lingyu Zhan, Alexandria Evans, Annabel Vreeker, Roel A. Ophoff, Neeltje E.M. van Haren

**Affiliations:** a Department of Child and Adolescent Psychiatry/Psychology, Erasmus University Medical Centre Rotterdam, Rotterdam, the Netherlands; b Semel Institute for Neuroscience and Human Behavior, University of California, Los Angeles, Los Angeles, California; c Developmental and Educational Psychology, Institute of Psychology, Leiden University, Leiden, the Netherlands; d Department of Psychology, Education and Child Studies, Erasmus University Rotterdam, Rotterdam, the Netherlands

**Keywords:** Bipolar, Familial risk, fMRI, Polygenic risk, Psychosis, Reward

## Abstract

**Background:**

Function of the striatal reward system has been implicated in both psychotic and bipolar disorders. We aimed to test whether ventral striatum (VS) activation during reward anticipation meets criteria of an endophenotype of these disorders. We hypothesized VS activation to be lower in patients than control participants, with nonaffected relatives in between. We also expected negative associations of VS activation with polygenic risk scores (PRSs) and with depressive and negative psychotic symptoms.

**Methods:**

One hundred twenty-seven patients, 66 control participants, and 51 first-degree relatives completed the monetary incentive delay task during functional magnetic resonance imaging. Diagnoses were confirmed with clinical interviewing, and PRSs for schizophrenia, bipolar disorder, and major depressive disorder were calculated from DNA testing of whole blood. Neural activation in the VS during reward anticipation was the main outcome. Linear mixed effects models tested 1) group differences (patients, relatives, control participants), 2) effects of manic, depressive, and positive and negative psychotic symptoms (accounting for group for depressive symptoms), and 3) effects of PRSs.

**Results:**

Groups did not differ significantly in VS activation during reward anticipation. Current symptom levels were not significantly related to VS activation, nor was there an interaction with group. PRSs were not significantly associated with VS activation. Findings were consistent after controlling for substance use and medication use.

**Conclusions:**

We did not find evidence that VS activation during reward anticipation meets criteria of an endophenotype of bipolar and psychotic disorders. Future research should examine associations between neural activation and symptom levels across diagnostic boundaries in a more symptomatic sample.

## Plain Language Summary

Dysfunction of the reward system in the brain has been implicated in both psychotic and bipolar disorders. We examined the function of a key region in this system during anticipation of rewards. We investigated differences between people with a psychotic or bipolar disorder, their first-degree family members, and control participants. We also looked at associations of brain function with genetic risk scores and with depressive, manic, and psychotic symptoms. Groups did not differ in neural activation during reward anticipation. Current symptom levels and genetic risk scores were not related to neural activation.

Schizophrenia (SZ) and bipolar disorder (BD) are highly heritable psychiatric disorders that share genetic risk and have clinical characteristics in common. For example, psychotic experiences are a core feature of SZ, but they also occur in the majority of patients with BD. Depressive symptoms are a core component of BD but are also reported in patients with SZ. Both disorders are characterized by alterations in reward-related brain function and behavior. For both disorders, an etiological theory exists that gives a key role to the striatal reward system (1,2). For SZ, this includes the idea that striatal dopamine dysregulation may impair the ability to distinguish signals indicating reward from those less indicative of reward, which could be expressed in impaired reward anticipation. For BD, this includes the idea of biased use of reward prediction errors, leading to disturbed reward anticipation.

Striatal and medial-frontal regions play a central role in the reward circuitry of the brain. During both the anticipation and feedback phases of reward processing, activation has been consistently documented in the striatum, especially the ventral striatum (VS) (3, 4, 5). The ventromedial prefrontal cortex (vmPFC)/ventral anterior cingulate cortex (vACC) and posterior cingulate cortex are active during the feedback phase specifically (3,4). The anterior insula responds to both reward and loss anticipation but not to reward feedback (3).

Neuroimaging studies have reported abnormal functioning in reward circuitry in individuals with SZ or BD during reward expectation and processing. A meta-analysis of 17 studies comparing patients with SZ with control participants reported hypoactivation in patients in the VS, dorsal ACC, midcingulate cortex, amygdala, precentral gyrus, and superior temporal gyrus compared with control participants (6). Striatum hypoactivation was stronger in studies where participants reported more negative symptoms but not positive symptoms and in studies with fewer second-generation antipsychotic users. Consistent with this link with negative symptoms, associations have been found between VS hypoactivation and depressive or anhedonic symptoms (7, 8, 9). A core component of these symptoms is lowered motivation, and VS signals are a driver of motivation. Interestingly, more polygenic risk for psychosis is related to more VS activation during reward anticipation and feedback (10). This was in healthy adolescents, though, and reward processing evolves during adolescent development. A whole-brain meta-analysis of 15 studies that compared individuals with BD to control participants revealed hypoactivation in the bilateral angular gyrus and right inferior frontal gyrus during reward anticipation (11). Hypoactivation in the VS was found for euthymic individuals with BD specifically (11).

Despite VS hypoactivation being reported across diagnoses and related to anhedonic and negative symptoms as described above, studies have tended to not cross diagnostic boundaries [with the exception of (12)]. Also, patient-control participant comparisons cannot inform us about the origins of any deviations in brain function; deviations in brain function could be a cause of the illness but also a consequence of the illness or medication use. For example, antipsychotics act by dampening the activity of dopamine, a key neurotransmitter in the striatum. In this regard, familial high-risk designs (i.e., studies in relatives of patients) are more informative.

Lower VS activation to rewards has been found repeatedly in first-degree relatives of patients with SZ (12, 13, 14), although null results have also been reported (15). Two studies found that this hypoactivation was related to negative symptoms (12,13). For relatives of individuals with BD, reduced VS activation has not been found consistently (16, 17, 18, 19), and group differences in other brain regions have been inconsistent (16, 17, 18, 19), but sample sizes tended to be small for these studies.

Taken together, this previous research raises the question of whether the blunting of VS activation during reward anticipation may serve well as a cross-diagnostic endophenotypic marker of SZ and BD. Alternatively, it could be a state-dependent driver of depressive and negative symptoms, as these symptoms also occur more often in first-degree relatives of patients with SZ and BD. Elucidating endophenotypes aids the development of animal models and can lead to a more biologically based classification of psychiatric phenomena (20). For example, animal models could include optogenetically manipulating VS neurons. If blunted VS activation is a state-dependent driver of depressive/negative symptoms, it can be tested as a treatment target for these symptoms (e.g., with brain stimulation or neurofeedback), which could benefit patients across multiple diagnostic categories.

Family designs allow for the examination of many criteria for endophenotypes. Gottesman and Gould (20) described these criteria as follows:

A. The endophenotype is associated with illness in the population.
B. The endophenotype is heritable.
C. The endophenotype is primarily state-independent (manifests in an individual whether or not illness is active).
D. Within families, endophenotype and illness co-segregate.
E. For identifying endophenotypes of diseases that display complex inheritance patterns, the endophenotype found in affected family members is found in nonaffected family members at a higher rate than in the general population.

We aimed to test these criteria in a sample of patients with a psychotic or bipolar disorder, their first-degree relatives, and control participants. To this end, we examined the following research questions:

1. Do patients with a psychotic or bipolar disorder, their first-degree relatives, and control participants differ in VS activation during reward anticipation?
2. How is VS activation during reward anticipation related to current depressive, manic, and psychotic symptoms?
3. How is VS activation during reward anticipation related to polygenic risk scores (PRSs) of BD, SZ, or depression?

We hypothesized that VS activation would be lower in patients than control participants (question 1, criterion A). Although not a direct test of heritability, negative associations with PRSs would establish a genetic contribution to neural activation during reward anticipation (question 3, criterion B). We hypothesized that VS activation would be negatively related to depressive and negative psychotic symptoms but that group differences would remain when taking current symptoms into account (question 2, criterion C). We also examined manic and positive psychotic symptoms to establish whether the association is specific to depressive/negative symptoms. Finally, we expected VS activation of nonaffected relatives to be in between that of patients and controls (question 1, criteria D and E). Of course, reward anticipation is only one element of reward processing. Research on familial high-risk groups’ neural activation during reward feedback (13,15,18,21) and loss anticipation and feedback (12,14,16,18,19) comes from small-*N* studies with inconsistent findings. Therefore, we cannot develop hypotheses regarding neural activation during these components of reward processing. However, we aimed to answer the 3 above-mentioned research questions for reward and loss feedback and loss anticipation in an exploratory way. Herein, we provide a more complete picture of reward processing in patients with BD or SZ, their relatives, and controls and a basis for generation of future hypotheses. For our preregistration, see: https://doi.org/10.17605/OSF.IO/WQBNE.

## Methods and Materials

### Participants and Procedure

Participants were recruited by recontacting participants of 2 previous studies [Dutch Bipolar Cohort (22,23) and Genetic Risk and Outcome of Psychosis (24)] and through patient organizations and psychiatric clinics in or near Rotterdam, the Netherlands. Inclusion criteria were: provided past consent to be recontacted for future studies (if recruited from previous studies); gave written informed consent for this study; mentally capacitated (no acute episode of psychosis or mania as determined by diagnostic interview, no guardianship measure, and no court authorization); and ≥18 years old. Exclusion criteria were: no demonstration of adequate understanding of the purpose, procedures, risks, benefits, and emergency contacts; magnetic resonance imaging (MRI) contraindications (including ferrous objects, pacemaker or other stimulatory devices, claustrophobia); history of closed head injury, neurological illness, or endocrine dysfunction; and neurological abnormalities as well as structural brain abnormalities that may interfere with the measurements, identified through neurodiagnostic evaluation. For group-specific criteria, see Group Status.

Participants provided written informed consent. Participation involved one visit of 4 to 5 hours, which included a clinical diagnostic interview; a 50-minute MRI session; 3 computer tasks; peripheral blood drawing; and measurement of height, weight, and blood pressure. After the visit, participants completed questionnaires at home and provided a fecal sample. Participants were allowed to opt out of specific elements or withdraw consent at any point. The procedures were approved by the Medical Ethics Committee of the Erasmus Medical Center. Data were collected between December 31, 2020 and September 8, 2024. For a power analysis, see the Supplement.

### Measures

#### Group Status

Diagnosis was confirmed for patients using the Comprehensive Assessment of Symptoms and History (CASH) interview (25). Relatives and control participants were assessed for psychopathology using the Mini-International Neuropsychiatric Interview (MINI) (26). In both cases, interviews were conducted by trained researchers, and diagnostic decisions were made based on consensus between 2 researchers and following DSM-5 criteria. Patients could have either BD type 1 (with or without psychotic features), BD type 2, BD not otherwise specified (NOS), SZ, schizoaffective disorder, schizophreniform disorder, delusional disorder, brief psychotic disorder, or psychotic disorder NOS. Relatives were required to have a first-degree family member with one of the diagnoses mentioned above but should not have had these diagnoses themselves. The family members with BD/SZ did not have to participate themselves if their diagnostic status was confirmed as part of the prior cohorts we recruited from (see Participants and Procedure). Relatives and control participants were not required to be free of all psychopathology. Group status was based on the results of the CASH and MINI; thus, for example, a participant could come in as a relative or control participant and be moved to the patient group when the MINI interview resulted in a diagnosis of a bipolar or psychotic disorder.

#### MRI Scan Parameters

MRI data were collected on a 3T GE Signa Premier scanner with 48-channel head coil at the Erasmus Medical Center in Rotterdam, the Netherlands. The task functional MRI (fMRI) was a T2∗-weighted echo planar imaging scan with 1.875 × 1.875 × 2.30-mm voxels, 304 volumes of 80 slices with 128 × 128 mm FOV, TR = 2.5 s, TE = 20 ms, and flip angle = 60. Multiband acceleration was used with phase acceleration of 2 and hyperband slice factor of 2. A T1-weighted scan was acquired for registration purposes with the following parameters: 0.47 × 0.47 × 0.8-mm voxels, 216 slices of 512 × 512 mm, TR = 7.1 ms, TE = 2.1 ms, flip angle = 12°.

#### Monetary Incentive Delay Task

**Figure 1:**
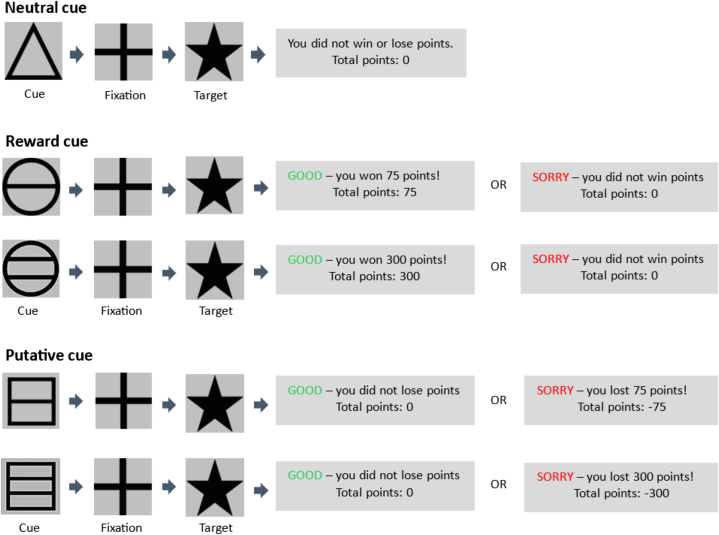
Schematic of each type of trial in the Monetary Incentive Delay task.

Figure 1 summarizes the task design. On each of 60 trials, a cue was presented signaling whether the subject could win or lose points or neither and the amount of points at stake. Participants responded as quickly as possible when a black star appeared on the screen. The star duration was individually titrated. Then participants received feedback on points lost or gained. For additional details, see the Supplement.

#### Symptom Measures

Patients completed the Altman Self-Rating Mania (ASRM) scale (27), the Inventory of Depressive Symptomatology (IDS) self-report (28), and the Positive and Negative Syndrome Scale (PANSS) (29). Relatives and control participants completed the IDS only as the ASRM and PANSS were developed for and have been validated in patients specifically. The ASRM is a 5-item self-report questionnaire that assesses manic or hypomanic symptoms during the past 7 days. The IDS is a 30-item self-report questionnaire on depressive symptoms during the past 7 days. For these 2 instruments, we used the total score. The PANSS is an interview and observational measure about psychotic symptoms in the past 7 days. We examined positive and negative symptoms from the PANSS separately [based on the 3-factor model (29)].

#### Polygenic Risk Scores

Blood samples were genotyped using procedures described in (30,31). Imputation was performed on the genotype data using Beagle version 5.5 (32) and 1000 Genomes Project phase 3 data (GRCh38) as the reference panel (33). Then, BP, SZ, and major depressive disorder (MDD) polygenic risk scores (PRSs) were calculated by first generating posterior single-nucleotide polymorphism effect size estimates using PRS with continuous shrinkage, a robust Bayesian framework known for its improved prediction accuracy (34), and the latest genome-wide association study summary statistics of the corresponding phenotypes (35, 36, 37), followed by individual-level PRS calculation using the “score” function in PLINK version 1.9 (38).

#### Covariates

Age and sex (male or female) were self-reported. The subjects’ own and highest parental education level were reported by participants and were included as covariates with the following categories: completed less than secondary education, secondary education, vocational tertiary education, professional tertiary education, university degree.

### Proposed Analyses

#### Processing of MRI Data

fMRIprep version 23 was used to preprocess the fMRI data. The processing of the T1-weighted images included correction for intensity nonuniformity and skull-stripping. Brain surface reconstruction and tissue segmentation were performed and used to refine the brain mask and improve registration of the fMRI data to the T1-weighted scan. Finally, spatial normalization to Montreal Neurological Institute (MNI) space was performed through nonlinear registration.

Functional data were motion corrected, slice timing corrected, and susceptibility distortion corrected using fieldmapless correction with symmetrical normalization. Functional scans were registered to the person’s T1-weighted space and normalized to the MNI template (ICBM 152 Nonlinear Asymmetrical template version 2009c) through coregistration to the T1-weighted image. All corrections and registrations were concatenated and applied in a single step. Finally, preprocessed functional data were smoothed using a 4-mm full-width at half maximum smoothing kernel.

Scans were visually inspected for artifacts. Extreme outliers in temporal signal to noise ratio (>4 SD from the sample mean) were excluded. In addition, scans containing >25% volumes with >0.8 mm relative framewise displacement were excluded.

First-level models were estimated in AFNI. For each scan, event-related models were set up following the general linear model, using a canonical hemodynamic response function, high-pass filtering of 100 seconds, and autocorrelation modeling. The first 4 to 8 volumes were removed to avoid non-steady state data; these were acquired before the task started by design. Motion parameters (translation along the 3 axes and rotation around the 3 axes) were added to these subject-level models as regressors of no interest. Volumes with more than 10% of brain voxels classified as outliers (calculated by 3dToutcount) were censored. Volumes with motion higher than 0.8 (Euclidean norm) were also censored.

For reward anticipation, trials were modeled as instantaneous starting at the time the cue appeared. The following 3 conditions were modeled: (both large and small) reward, (both large and small) loss, and neutral trials. Contrasts were created of reward versus neutral (i.e., reward anticipation) and loss versus neutral (i.e., loss anticipation). For reward feedback, trials were modeled starting at the time feedback appeared, with a duration of 2000 ms. The following 5 conditions were modeled: (large or small) reward received, (large or small) loss avoided, (large or small) reward not received, (large or small) loss not avoided, and neutral trials. Contrasts were created of reward received versus reward not received (i.e., reward feedback) and loss not avoided versus loss avoided (i.e., loss feedback).

#### Region of Interest Definition and Activation Extraction

The ventral striatal regions of interest (ROIs) were created by placing 8-mm spheres around the peaks reported in the meta-analysis of reward anticipation by Chen *et al.* (3) (x, y, z = −10, 8, −4 and 10, 10, −2). Anatomically this includes the nucleus accumbens and ventral caudate. Average activation is extracted for the contrasts mentioned in the previous section. For this, we used AFNI’s 3dmaskave, and activation in the left and right hemisphere were averaged before being entered in statistical models. We also created (unthresholded) whole-brain statistical maps and shared these on https://identifiers.org/neurovault.collection:22083 so that they can be used for meta-analysis. The whole-brain analyses had the same statistical modeling as described below for the ROI analyses, but they were run with AFNI’s 3dLMER.

#### Statistical Modeling of Confirmatory Research Questions

Assumptions were checked. We ran linear mixed effects models in R (version 4.1.1) with random intercepts to adjust for family relationships between participants. When the variance of this random intercept was 0, we switched to linear regression (i.e., the same model without the random intercept). Age, sex, and education level were added as covariates in all models. We applied a *p* value threshold of .05 after false discovery rate (FDR) correction across the 8 confirmatory analyses. R formulas were as follows:

• Research question 1: VS activation ∼ group + age + sex + own_education + parent_education + (1 | family_id).
• Research question 2: VS activation ∼ group + depressive_symptoms + depressive_symptoms × group + age + sex + own_education + parent_education + (1| family_id).

Manic and psychotic symptoms were only available in patients. Therefore, we set up linear regression with neural activation as the outcome and either manic, positive, or negative psychotic symptoms as predictor.

• Research question 3: VS activation ∼ polygenic_risk + age + sex + own_education + parent_education + (1 | family_id). Separate models were run for BD, SZ, and depression risk scores.

#### Statistical Modeling of Exploratory Analyses

We repeated the above-described models for neural activation during loss anticipation, reward feedback, and loss feedback. In addition, we added the anterior insula and vmPFC/vACC as ROIs (3,4). The vmPFC/vACC is active during reward feedback (3,4) and the anterior insula reward and loss anticipation and loss feedback (3), and altered activation in these regions has been reported in either patients with SZ or BD (6,11). The anterior insula ROI was based on the Automated Anatomical Labeling atlas (setting to border at y = 10 for anterior part) and the vmPFC/vACC ROI was based on the Brainnetome atlas (A32sg left and right). We report *p* values before and after multiple comparisons correction using FDR correction across these exploratory analyses within each domain of reward processing (3 ROIs × 9 *p* values = 27 analyses per domain).

Finally, we ran sensitivity analyses including the following covariates all at once: nicotine intake per day, coffee intake per day, alcohol intake during the last month, and any recreational drug use (yes/no) during the last month. Substance intake was not restricted prior to or during the visit. For the analyses that only include patients (i.e., analyses on manic and psychotic symptoms), we also included medication type in this sensitivity analysis, with variables for lithium, first-generation antipsychotics, second-generation antipsychotics, antidepressants, and other psychotropic medication.

## Results

### Demographics

Two hundred fifty-two participants completed the fMRI scan. Three participants were excluded due to too many nonresponses on the Monetary Incentive Delay (MID) task, 3 participants due to head motion on the scan, and 2 participants due to artifacts on the scan detected with visual inspection. This led to a final sample of 244 participants. Descriptive information about the sample can be found in Table 1. Of the patients, 92 had BD type 1, 5 had another BD, 10 had schizoaffective disorder, 3 had delusional disorder, 2 had brief psychotic disorder, and 14 had SZ. See Table 1 for other diagnoses by group.

**Table 1:** Descriptive Information About the Sample and Key Variables

	Patients, *n* = 127	Relatives, *n* = 51	Control Participants, *n* = 66	Group Differences
Age, Years	52.0 (11.5)	53.5 (11.1)	52.3 (12.5)	*F*_2,241_ = 0.3, *p* = .74
Sex, Female	52%	57%	46%	χ^2^_2_ = 1.6, *p* = .50
Education Level, Own Education/Parental Education				
Less than secondary	3%/6%	0%/11%	0%/9%	Own: χ^2^_2_ = 12.5, *p* = .002, Secondary P > R&C Parental: χ^2^_2_ = 3.7, *p* = .16
Secondary	23%/12%	6%/4%	7%/13%	
Vocational tertiary	20%/35%	16%/26%	20%/41%	
Professional tertiary	30%/21%	52%/41%	36%/30%	
University	25%/26%	26%/17%	38%/7%	
Depression Symptoms	14.0 (10.2)	7.7 (6.8)	5.8 (5.2)	*F*_2,222_ = 22.1, *p* < .001, P > R&C
Mania Symptoms	1.9 (2.4)	NA	NA	NA
Positive Psychotic Symptoms	9.4 (3.4)	NA	NA	NA
Negative Psychotic Symptoms	10.0 (3.0)	NA	NA	NA
Polygenic Risk Score Bipolar Disorder	0.34 (0.09)	0.27 (0.09)	0.22 (0.11)	*F*_2,148_ = 21.1, *p* < .001, P > R > C
Polygenic Risk Score Schizophrenia	0.94 (0.23)	0.86 (0.25)	0.74 (0.30)	*F*_2,148_ = 8.5, *p* < .001, P > C
Polygenic Risk Score Major Depressive Disorder	0.20 (0.09)	0.19 (0.09)	0.18 (0.10)	*F*_2,148_ = 0.24, *p* = .79
Money Won on the MID Task, €	12.5 (9.0)	15.2 (6.3)	14.0 (6.2)	*F*_2,241_ = 2.4, *p* = .09
Accuracy Difference Between Reward and Neutral Trials on the MID Task	9.1 (16.7)	13.3 (20.0)	6.6 (16.5)	*F*_2,241_ = 2.2, *p* = .12
Reaction Time Difference Between Reward and Neutral Trials on the MID Task	−6.9 (16.9)	−2.3 (16.1)	−4.4 (13.2)	*F*_2,241_ = 1.7, *p* = .19
Current Medication Use				
Lithium	42%	0%	0%	χ^2^_2_ = 61.6, *p* < .001, P > R&C
Antidepressants	26%	14%	3%	χ^2^_2_ = 16.6, *p* < .001, P > R > C
First-generation antipsychotics	2%	0%	0%	χ^2^_2_ = 2.8, *p* = .25
Second-generation antipsychotics	42%	0%	0%	χ^2^_2_ = 62.4, *p* < .001, P > R&C
Other psychotropic medication	32%	2%	3%	χ^2^_2_ = 35.1, *p* < .001, P > R&C
Diagnoses, Past and Present Combined				
Bipolar disorders	76%	NA	NA	NA
Schizoaffective	8%	NA	NA	NA
Schizophrenia and other psychotic disorders	15%	NA	NA	NA
Depressive disorders	1%	31%	23%	χ^2^_2_ = 35.1, *p* < .001, P < R&C
Anxiety disorders	2%	29%	15%	χ^2^_2_ = 27.9, *p* < .001, P < R&C
ADHD	0%	4%	11%	χ^2^_2_ = 13.8, *p* = .001, P < R&C
Problematic substance use, excluding smoking	33%	22%	15%	χ^2^_2_ = 7.9, *p* = .02, P > C
Other diagnoses	0%	10%	8%	χ^2^_2_ = 11.7, *p* = .003, P < R&C
Any Drug Use Past Month, Yes	7%	2%	3%	χ^2^_2_ = 2.7, *p* = .25
Alcohol Intake, Glasses per Week Past Month	4.3 (6.9)	7.5 (12.1)	4.3 (6.1)	*F*_2,241_ = 3.2, *p* = .04, R > P
Coffee Intake, Cups per Day	3.5 (2.8)	3.1 (2.3)	3.7 (3.5)	*F*_2,240_ = 0.4, *p* = .64
Nicotine Intake, Cigarettes per Day	2.7 (6.7)	0.9 (3.0)	0.6 (2.5)	*F*_2,241_ = 4.3, *p* = .01, P > C

Numbers indicate mean (SD) or percentages. The right-most column indicates overall group differences, and pairwise comparisons with patients (P), relatives (R), and control participants (C).

ADHD, attention-deficit/hyperactivity disorder; MID, Monetary Incentive Delay; NA, not applicable.

Age, sex, and parental education did not differ between the groups, whereas participants’ own education did (Table 1). Also, depression symptom scores were higher in patients than in the other 2 groups. PRSs for BD and SZ but not for MDD were higher in patients than in relatives and controls. The PRSs for BD and SZ in relatives were in between that of patients and controls.

Groups won a similar amount of money on the MID task (Table 1). The accuracy difference and reaction time difference between reward and neutral trials did not differ significantly between groups. The accuracy difference between reward and neutral trials was significantly different from 0 in each group (*t*_126_ = 6.14, *p* < .001; *t*_50_ = 4.75, *p* < .001; *t*_65_ = 3.25, *p* = .002), so participants in all groups were more likely to press in time when a reward was offered (i.e., task manipulation worked). Reward anticipation elicited positive activation of the VS across the sample, as expected (see Table S1).

### Group Differences (Research Question 1)

**Figure 2:**
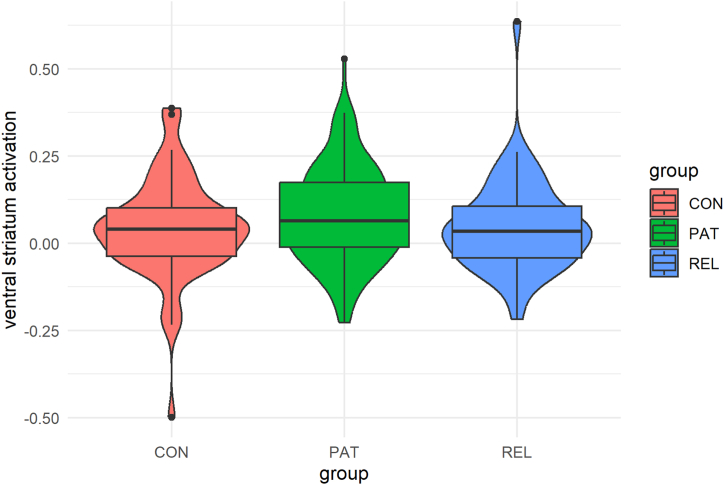
Ventral striatum activation during reward anticipation in control participants (CON), patients (PAT), and first-degree relatives of patients (REL).

Groups did not differ significantly in VS activation during reward anticipation (Figure 2, Table 2, and Table S1).

**Table 2:** Results of Analyses of Reward Anticipation

	Ventral Striatum				Anterior Insula				vmPFC/vACC			
	b	SE	*t*	*p*	b	SE	*t*	*p*	b	SE	*t*	*p*
Group Differences												
PAT-CON	0.23	0.02	1.46	.15	−0.003	0.02	−0.21	.84	0.04	0.02	0.16	.87
REL-CON	0.04	0.03	0.19	.85	−0.03	0.02	−1.72	.09	0.003	0.02	−0.96	.34
Depression Symptoms	−0.002	0.004	−0.63	.53	−0.003	0.002	−1.04	.30	−0.003	0.003	−0.93	.35
Mania Symptoms	−0.004	0.006	−0.69	.49	−0.004	0.004	−0.97	.34	−0.000	0.006	−0.001	.99
Positive Psychotic Symptoms	−0.000	0.005	−0.008	.99	−0.001	0.003	−0.39	.70	−0.004	0.004	−0.84	.41
Negative Symptoms	0.004	0.004	0.92	.36	0.002	0.003	0.56	.58	0.002	0.004	0.48	.63
SZ Polygenic Risk Score	0.04	0.05	0.86	.39	−0.005	0.03	−0.16	.87	0.05	0.03	1.34	.18
BD Polygenic Risk Score	0.15	0.12	1.28	.20	−0.02	0.08	−0.29	.77	0.13	0.09	1.48	.14
MDD Polygenic Risk Score	−0.04	0.14	−0.26	.80	0.05	0.09	0.52	.60	−0.02	0.10	−0.17	.87

Listed *p* values are before FDR correction; after correction, all *p* values were >.50 (not shown).

BD, bipolar disorder; CON, control participants; FDR, false discovery rate; MDD, major depressive disorder; PAT, patients; REL, relatives; SZ, schizophrenia; vACC, ventral anterior cingulate cortex; vmPFC, ventromedial prefrontal cortex.

### Symptoms (Research Question 2)

**Figure 3:**
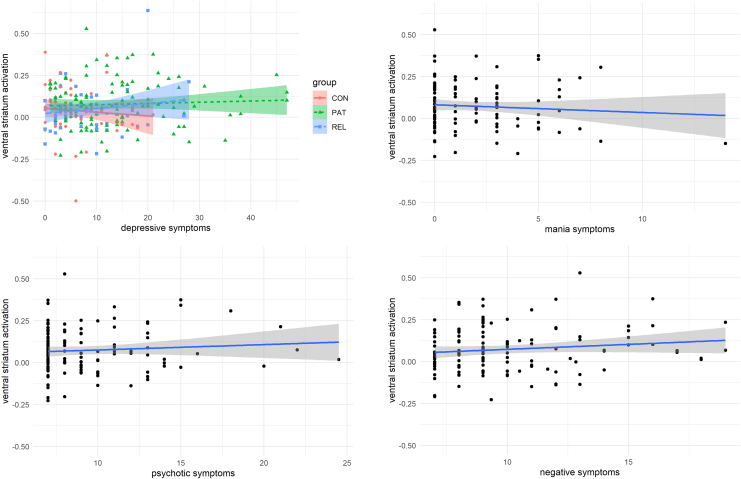
Ventral striatum activation during reward anticipation in relation to depressive symptoms in all groups (control participants [CON], patients [PAT], and first-degree relatives of patients [REL]) and to mania symptoms, psychotic symptoms, and negative symptoms in patients.

Depressive symptoms were not significantly related to VS activation during reward anticipation, and there was no interaction with group. Mania symptoms, positive psychotic symptoms, and negative symptoms were also not significantly related to VS activation during reward anticipation (Figure 3, Table 2, and Tables S2–S5).

### PRSs (Research Question 3)

One hundred fifty-one participants provided blood samples and gave permission for these samples to be analyzed for DNA at the laboratory of the Center for Neurobehavioral Genetics at the University of California at Los Angeles (74 patients, 35 relatives, and 42 control participants). The PRSs were not significantly related to VS activation during reward anticipation (Table 2 and Tables S6–S8).

### Sensitivity Analyses and Exploratory Analyses

We repeated the analysis for research question 1 after adding the substance use variables. This did not change the significance of the results, and none of the substance use variables were associated with VS activation. We repeated the analysis for depressive symptoms (research question 2) after adding the substance use variables, and this also did not change the significance of the results. Finally, we repeated the analyses for mania and psychosis symptoms (research question 2) in patients after adding the substance use variables and medication variables, and this also did not change the significance of the results. The substance use and medication variables were not significantly related to VS activation.

See Table 2, Table 3, Table 4, Table 5 for the results of the exploratory analyses in the anterior insula and vmPFC/vACC during reward anticipation, reward feedback, loss anticipation, and loss-avoidance feedback.

**Table 3:** Results of Exploratory Analyses of Reward Feedback

	Ventral Striatum				Anterior Insula				vmPFC/vACC			
	b	SE	*t*	*p*	b	SE	*t*	*p*	b	SE	*t*	*p*
Group Differences												
PAT-CON	0.02	0.03	1.49	.13	0.02	0.02	0.99	.33	0.04	0.03	1.33	.19
REL-CON	0.05	0.04	2.17	.03∗	−0.004	0.03	−0.17	.87	0.01	0.04	0.34	.74
Depression Symptoms	0.005	0.005	1.05	.30	−0.002	0.004	−0.58	.56	0.001	0.005	0.24	.81
Mania Symptoms	−0.000	0.009	−0.05	.96	−0.006	0.007	−0.81	.42	0.003	0.01	0.28	.78
Positive Psychotic Symptoms	0.005	0.007	0.70	.49	0.004	0.006	0.72	.47	0.002	0.007	0..25	.80
Negative Symptoms	0.004	0.005	0.86	.39	−0.006	0.005	−1.06	.29	0.002	0.007	0.32	.75
SZ Polygenic Risk Score	0.03	0.06	0.44	.66	0.11	0.04	2.37	.02∗	0.16	0.06	2.62	.01∗
BD Polygenic Risk Score	0.07	0.15	0.45	.65	−0.009	0.12	−0.08	.94	0.03	0.16	0.17	.87
MDD Polygenic Risk Score	−0.001	0.18	−0.003	1	0.14	0.14	1.01	.31	0.10	0.19	0.52	.60

Listed *p* values are before FDR correction; after correction, all *p* values were >.26 (not shown).

∗*p* Values <.05 before correction.

BD, bipolar disorder; CON, control participants; FDR, false discovery rate; MDD, major depressive disorder; PAT, patients; REL, relatives; SZ, schizophrenia; vACC, ventral anterior cingulate cortex; vmPFC, ventromedial prefrontal cortex.

**Table 4:** Results of Exploratory Analyses of Loss Anticipation

	Ventral Striatum				Anterior Insula				vmPFC/vACC			
	b	SE	*t*	*p*	b	SE	*t*	*p*	b	SE	*t*	*p*
Group Differences												
PAT-CON	0.02	0.02	1.20	.23	−0.003	0.01	−0.26	.79	−0.02	0.02	−0.92	.36
REL-CON	0.02	0.02	0.88	.38	−0.02	0.02	−1.05	.30	−0.03	0.02	−1.27	.21
Depression Symptoms	−0.002	0.003	−0.80	.42	−0.004	0.002	−2.04	.04∗	−0.002	0.003	−0.75	.46
Mania Symptoms	−0.000	0.006	−0.04	.97	0.005	0.004	1.21	.23	0.002	0.006	0.37	.71
Positive Psychotic Symptoms	−0.005	0.005	−1.09	.28	−0.001	0.003	−0.19	.85	−0.004	0.005	−0.81	.42
Negative Symptoms	0.000	0.004	0.03	.97	0.000	0.003	0.12	.90	−0.003	0.005	−0.64	.52
SZ Polygenic Risk Score	0.09	0.04	2.17	.03∗	0.02	0.03	0.59	.56	0.04	0.04	1.02	.31
BD Polygenic Risk Score	0.17	0.10	1.66	.10	−0.03	0.07	−0.50	.62	0.07	0.10	0.73	.47
MDD Polygenic Risk Score	0.03	0.12	0.24	.81	0.05	0.08	0.67	.51	0.004	0.11	0.03	.98

Listed *p* values are before FDR correction; after correction, all *p* values were >.50 (not shown). ∗*p* Values were <.05 before correction.

BD, bipolar disorder; CON, control participants; FDR, false discovery rate; MDD, major depressive disorder; PAT, patients; REL, relatives; SZ, schizophrenia; vACC, ventral anterior cingulate cortex; vmPFC, ventromedial prefrontal cortex.

**Table 5:** Results of Exploratory Analyses of Loss Avoidance Feedback

	Ventral Striatum				Anterior Insula				vmPFC/vACC			
	b	SE	*t*	*p*	b	SE	*t*	*p*	b	SE	*t*	*p*
Group Differences												
PAT-CON	0.02	0.03	0.80	.42	0.03	0.02	1.41	.16	0.009	0.03	0.26	.80
REL-CON	0.008	0.03	0.26	.80	−0.02	0.03	−0.60	.55	−0.06	0.04	−1.56	.12
Depression Symptoms	0.005	0.004	1.16	.25	0.001	0.003	0.29	.78	−0.001	0.005	−0.19	.85
Mania Symptoms	−0.007	0.008	−0.89	.38	−0.001	0.007	−0.10	.92	−0.008	0.01	−0.68	.50
Positive Psychotic Symptoms	0.002	0.006	0.28	.78	0.002	0.005	0.30	.77	0.004	0.009	0.43	.67
Negative Symptoms	−0.000	0.006	−0.04	.96	0.001	0.005	0.24	.81	0.005	0.008	0.64	.53
SZ Polygenic Risk Score	0.10	0.05	1.97	.05∗	0.07	0.04	1.71	.09	0.09	0.07	1.38	.17
BD Polygenic Risk Score	0.12	0.14	0.86	.39	0.02	0.11	0.16	.87	0.08	0.17	0.42	.68
MDD Polygenic Risk Score	−0.002	0.17	−0.01	.99	−0.11	0.14	−0.80	.43	−0.07	0.21	−0.35	.73

Listed *p* values are before FDR correction; after correction all *p* values were >.50.

∗*p* Values <.05 before correction.

BD, bipolar disorder; CON, control participants; FDR, false discovery rate; MDD, major depressive disorder; PAT, patients; REL, relatives; SZ, schizophrenia; vACC, ventral anterior cingulate cortex; vmPFC, ventromedial prefrontal cortex.

## Discussion

We aimed to test whether VS activation during reward anticipation meets criteria of an endophenotype of psychotic disorders and BDs. To this end, we examined differences between patients, their first-degree relatives, and control participants; analyzed how VS activation is related to current depressive, manic, and psychotic symptoms; and examined how it is related to PRSs of BD, SZ, and depression. Groups did not differ in VS activation during reward anticipation. Current symptom levels were not related to VS activation, and there was no interaction with group. PRSs were not associated with VS activation. Findings were consistent after controlling for substance use and medication use.

These findings imply that we cannot confirm that VS activation during reward anticipation meets criteria of an endophenotype of psychotic and bipolar disorders. Contradictory to our hypotheses and previous literature (6,11), patients and control participants did not differ in VS activation. Reward anticipation did elicit positive activation of the VS across the sample, as expected. These neural findings are consistent with our behavioral findings; participants in all groups were more likely to press in time when a reward was offered, which suggests that the task manipulation worked, but groups did not differ in task performance. We had enough power to detect a medium effect size, and the above-cited meta-analyses found medium to large effects (6,11). One difference between our study and the previous literature is that our sample is older on average and therefore has a relatively long history of illness and medication use. However, current medication use did not make a difference to our findings, and prior studies did not find effects to be dependent on age or illness duration. Our participants with a psychotic or bipolar disorder were also often in a stable phase of illness and low in symptoms (see Strengths and Limitations), suggesting that the VS deactivation may be limited to patients with a more severe course of illness or in a current episode.

Previous findings in individuals at familial risk, especially relatives of patients with BD (16, 17, 18, 19), have been inconsistent, and therefore our findings on relatives versus control participants do not necessarily contradict the literature. In addition, there were no associations between polygenic risk and VS activation, although the group differences in polygenic risk for BD and SZ confirm expected risk patterns (highest in patients, lowest in control participants) and confirm the overlap between the disorders. A meta-analysis comparing relatives and control participants would be useful to make more definitive conclusions, but taking these findings and the previous literature together, the evidence does not suggest that VS hypoactivation during reward anticipation is a consequence of familial risk for bipolar or psychotic disorders.

An alternative hypothesis that we proposed in the introduction is that VS hypoactivation could be a state-dependent driver of depressive and negative symptoms. These symptoms are common across bipolar and psychotic disorders. However, we did not find current symptom levels to be related to VS activation. The lack of associations with symptoms might be explained by the low levels of current symptoms in the sample, resulting in limited variance in the symptom variables. It would be useful to repeat these analyses in a cross-diagnostic sample with higher current symptom levels.

### Strengths and Limitations

Strengths of this study include the inclusion of patients, first-degree relatives, and control participants and the cross-diagnostic approach to examining symptoms and genetic risk. However, the current findings have to be considered in light of several limitations. First, the (self-)selection bias might have led to a relatively motivated and well-educated sample, with patients who were often high-functioning and low in current symptoms. Partially, this is difficult to avoid, as an acute psychotic or manic episode would interfere with participants’ ability to provide informed consent, and those with strong anhedonic symptoms may not be motivated to take part in research. This sampling could have led to underestimation of group differences and limited variance in the symptom measures. Furthermore, we did not distinguish between diagnostic categories within the group with mood–psychosis spectrum disorders. This was by design as we took a dimensional approach, focusing on symptoms that occur across the diagnostic categories. In addition, we were interested in mechanisms in which familial risk expresses itself, and familial risk also occurs heterotypically, i.e., across diagnostic boundaries. However, this means that we cannot say whether neural activation differed by diagnostic category. Finally, in our power analyses, we accounted for multiple testing, and thus it could be argued that our study, despite having a relatively large sample size compared with current literature, is still underpowered, although the main analyses also showed no findings with *p* < .05 before multiple comparisons correction.

### Conclusions

We did not find evidence that VS activation during reward anticipation meets criteria of an endophenotype of bipolar and psychotic disorders. Future research should examine associations between neural activation and symptom levels across diagnostic boundaries in a more symptomatic sample.

## Supplementary Material

Supplement Methods and Tables S1–S8

Key Resources Table
