# Supplementary material for: Reward-Related Brain Function as an Endophenotype in the Mood-Psychosis Spectrum
Source: Biol Psychiatry Glob Open Sci. 2026 Jan 21;6(3):100692. doi: 10.1016/j.bpsgos.2026.100692 (PMC13080669; doi:10.1016/j.bpsgos.2026.100692)
Supplement: Supplement Methods and Tables S1–S8 [file mmc1.pdf]

SUPPLEMENTARY INFORMATION

Reward-Related Brain Function as an Endophenotype in the Mood-Psychosis Spectrum

Barendse *et al.*

Supplementary materials

Power analysis

Power analyses for linear mixed effect models assume more knowledge about the variances of your variables and expected differences between groups than we have, and assume equal cluster sizes (in our cases family sizes). Therefore, we conducted a power analysis for an ANCOVA (model 1 and 2) and linear regression (model 3), which are the equivalent models without the random intercept by family ID. The results show that we need 206 participants (model 1 and 2) or 108 participants (model 3) for 90% power and medium effect size, demonstrating that we have sufficient numbers of participants for the three models.

Detailed description MID fMRI task

On each trial of the MID task, a cue is presented for 500ms signaling whether the subject could win or lose points (circle=win trial and square=lose trial) or not (triangle=neutral trial). For the win and lose trials, one horizontal line in the circle or square means 75 points are at stake, and two horizontal lines mean 300 points are at stake. Immediately after the cue, a fixation cross is presented for a variable time (6 s minus cue duration minus star duration). Next, participants respond as quickly as possible when a target stimulus (black star) appears on the screen. Participants respond by pressing on the button box with their dominant hand. Subsequent feedback (2000ms) indicates if performance was correct (text in green font) or incorrect (text in red font), and whether they earned points, lost points, or nothing happened on that trial (indicated as + 75/300, - 75/300, or +0, respectively), and their cumulative total at that moment. For both potentially rewarding and non-rewarding trials, subjects have to respond to the target stimulus within a specific time limit (equal to the target duration). The target duration is individually titrated as the average of the 16th and 17th slowest response time of 24 baseline trials prior to the start of the experiment, and updated after every trial (if the proportion correct is below 66%, add 20ms; if the proportion correct is 66% or above, reduce by 10ms; as long as it stays within 150-400ms). The experiment contains 60 trials, 12 of each condition. The code and stimuli of the task are freely available at DOI 10.17605/OSF.IO/2WY7U.

Participants also played a computer version of the MID and the order of the in-scanner and outside-scanner version was randomized across participants. The participants were told that their performance on the MID and computer tasks would influence the amount of reward received, but in reality they received a fixed amount at the end of all assessments. They were debriefed about this at the end of the session. To assure that participants were paying attention, we excluded participants with ≥30% non-responses (n=3).

STable 1. Full model results of the main analysis of group differences

**Group differences**	estimate	standard error	df	t	p
intercept	0.230	0.106	195.19	2.166	0.03
group patients vs controls	0.036	0.024	197.99	1.463	0.15
group relatives vs controls	0.005	0.029	198.00	0.186	0.85
sex male vs female	-0.035	0.020	197.18	-1.757	0.08
age	-0.002	0.001	191.59	-2.432	0.02
education_level professional tertiary	-0.065	0.084	196.84	-0.769	0.44
education_level secondary	-0.074	0.086	197.21	-0.857	0.39
education_level university	-0.074	0.084	197.35	-0.878	0.38
education_level vocational tertiary	-0.026	0.087	197.47	-0.296	0.77
education_level_parents professional tertiary	0.009	0.041	182.71	0.225	0.82
education_level_parents secondary	-0.010	0.047	187.68	-0.21	0.83
education_level_parents university	0.019	0.043	157.88	0.438	0.66
education_level_parents vocational tertiary	0.004	0.039	171.61	0.099	0.92

STable 2. Full model results of the main analysis of depressive symptoms

**Depressive symptoms**	estimate	standard error	df	t	p
intercept	0.239	0.110	191.18	2.167	0.03
IDS total score	-0.002	0.004	192.79	-0.628	0.53
group patients vs controls	0.014	0.038	193.83	0.374	0.71
group relatives vs controls	-0.036	0.044	192.95	-0.808	0.42
age	-0.002	0.001	188.28	-2.438	0.02
sex male vs female	-0.032	0.020	193.84	-1.585	0.11
education_level professional tertiary	-0.063	0.085	192.51	-0.737	0.46
education_level secondary	-0.079	0.087	192.87	-0.912	0.36
education_level university	-0.070	0.085	192.93	-0.821	0.41
education_level vocational tertiary	-0.025	0.087	192.95	-0.288	0.77
education_level_parents professional tertiary	0.013	0.042	181.11	0.318	0.75
education_level_parents secondary	-0.010	0.047	185.64	-0.212	0.83
education_level_parents university	0.019	0.044	162.00	0.437	0.66
education_level_parents vocational tertiary	0.010	0.039	172.34	0.266	0.79
IDS total * group patients vs controls	0.003	0.004	193.70	0.746	0.46
IDS total * group relatives vs controls	0.006	0.005	193.85	1.194	0.23

STable 3. Full model results of the main analysis of mania symptoms in patients

**Mania symptoms**	estimate	standard error	df	t	p
intercept	0.343	0.129	91	2.66	0.009
Altman mania score	-0.004	0.006	91	-0.69	0.49
age	-0.003	0.001	91	-2.28	0.02
sex male vs female	-0.025	0.029	91	-0.88	0.38

education_level professional tertiary	-0.068	0.09	91	-0.794	0.43
education_level secondary	-0.085	0.088	91	-0.97	0.33
education_level university	-0.083	0.086	91	-0.96	0.34
education_level vocational tertiary	-0.004	0.09	91	-0.042	0.97
education_level_parents professional tertiary	-0.001	0.07	91	-0.022	0.98
education_level_parents secondary	-0.078	0.068	91	-1.13	0.26
education_level_parents university	-0.012	0.065	91	-0.19	0.85
education_level_parents vocational tertiary	-0.026	0.06	91	-0.434	0.67

STable 4. Full model results of the main analysis of positive psychotic symptoms in patients

**Psychotic symptoms**	estimate	standard error	df	t	p
intercept	0.363	0.139	92	2.601	0.01
PANSS positive score	0.000	0.005	92	-0.008	0.99
age	-0.004	0.001	92	-2.673	0.01
sex male vs female	-0.039	0.027	92	-1.418	0.16
education_level professional tertiary	-0.055	0.085	92	-0.652	0.52
education_level secondary	-0.060	0.086	92	-0.699	0.49
education_level university	-0.073	0.085	92	-0.855	0.39
education_level vocational tertiary	-0.001	0.089	92	-0.016	0.99
education_level_parents professional tertiary	-0.022	0.063	92	-0.359	0.72
education_level_parents secondary	-0.087	0.067	92	-1.292	0.20
education_level_parents university	-0.016	0.064	92	-0.249	0.80
education_level_parents vocational tertiary	-0.044	0.058	92	-0.765	0.45

STable 5. Full model results of the main analysis of negative psychotic symptoms in patients

**Negative symptoms**	estimate	standard error	df	t	p
intercept	0.319	0.135	92	2.371	0.02
PANSS negative score	0.004	0.005	92	0.920	0.36
age	-0.003	0.001	92	-2.528	0.01
sex male vs female	-0.041	0.027	92	-1.504	0.14
education_level professional tertiary	-0.064	0.084	92	-0.763	0.45
education_level secondary	-0.076	0.087	92	-0.880	0.38
education_level university	-0.079	0.084	92	-0.941	0.35
education_level vocational tertiary	-0.016	0.090	92	-0.176	0.86
education_level_parents professional tertiary	-0.021	0.062	92	-0.338	0.74
education_level_parents secondary	-0.092	0.067	92	-1.380	0.17
education_level_parents university	-0.017	0.063	92	-0.267	0.79
education_level_parents vocational tertiary	-0.041	0.057	92	-0.714	0.48

STable 6. Full model results of the main analysis of schizophrenia polygenic risk scores

**PRS SZ**	estimate	standard error	df	t	p
intercept	0.124	0.186	113.82	0.667	0.51
PRS schizophrenia	0.040	0.047	116.46	0.858	0.39
age	-0.002	0.001	114.53	-1.945	0.05
sex male vs female	-0.018	0.026	115.82	-0.700	0.49
education_level professional tertiary	0.033	0.152	114.13	0.215	0.83
education_level secondary	0.063	0.153	115.29	0.413	0.68
education_level university	0.005	0.151	114.37	0.032	0.97
education_level vocational tertiary	0.081	0.153	114.96	0.525	0.60
education_level_parents professional tertiary	0.011	0.053	112.58	0.204	0.84
education_level_parents secondary	-0.002	0.059	111.70	-0.039	0.97
education_level_parents university	0.043	0.054	110.99	0.793	0.43
education_level_parents vocational tertiary	-0.033	0.048	108.27	-0.691	0.49

STable 7. Full model results of the main analysis of bipolar disorder polygenic risk scores

**PRS BD**	estimate	standard error	df	t	p
intercept	0.127	0.178	113.90	0.717	0.47
PRS bipolar disorder	0.152	0.119	115.97	1.276	0.20
age	-0.002	0.001	114.48	-1.914	0.06
sex male vs female	-0.019	0.026	115.81	-0.715	0.48
education_level professional tertiary	0.019	0.149	114.57	0.126	0.90
education_level secondary	0.045	0.151	115.76	0.296	0.77
education_level university	-0.002	0.149	114.64	-0.014	0.99
education_level vocational tertiary	0.062	0.151	115.31	0.411	0.68
education_level_parents professional tertiary	0.011	0.052	112.39	0.214	0.83
education_level_parents secondary	-0.007	0.059	112.25	-0.120	0.90
education_level_parents university	0.041	0.054	111.06	0.751	0.45
education_level_parents vocational tertiary	-0.037	0.048	108.22	-0.761	0.45

STable 8. Full model results of the main analysis of major depressive disorder polygenic risk scores

**PRS MDD**	estimate	standard error	df	t	p
intercept	0.189	0.176	114.09	1.074	0.29
PRS MDD	-0.037	0.143	114.30	-0.257	0.80
age	-0.002	0.001	114.41	-1.986	0.05
sex male vs female	-0.018	0.026	116.16	-0.689	0.49
education_level professional tertiary	0.010	0.150	114.47	0.064	0.95
education_level secondary	0.045	0.152	115.73	0.294	0.77

education_level university	-0.013	0.149	114.56	-0.090	0.93
education_level vocational tertiary	0.065	0.152	115.32	0.425	0.67
education_level_parents professional tertiary	0.008	0.053	112.42	0.151	0.88
education_level_parents secondary	0.001	0.059	111.55	0.010	0.99
education_level_parents university	0.041	0.055	110.15	0.760	0.45
education_level_parents vocational tertiary	-0.035	0.049	107.74	-0.713	0.48
